# Induction of mycoplasmal pneumonia in experimentally infected pigs by means of different inoculation routes

**DOI:** 10.1186/s13567-016-0340-2

**Published:** 2016-05-09

**Authors:** Beatriz Garcia-Morante, Joaquim Segalés, Sergio López-Soria, Ana Pérez de Rozas, Henrike Maiti, Teresa Coll, Marina Sibila

**Affiliations:** IRTA, Centre de Recerca en Sanitat Animal (CReSA, IRTA-UAB), Campus de la Universitat Autònoma de Barcelona, 08193 Bellaterra, Spain; Boehringer Ingelheim España S.A, Carrer Prat de la Riba, 50, 08174 Sant Cugat del Vallès, Spain; UAB, Centre de Recerca en Sanitat Animal (CReSA, IRTA-UAB), Campus de la Universitat Autònoma de Barcelona, 08193 Bellaterra, Spain; Departament de Sanitat i Anatomia Animals, Facultat de Veterinària, UAB, 08193 Bellaterra, Spain; Boehringer Ingelheim Veterinary Research Center GmbH & Co, BemeroderStraße 31, 30559 Hannover, Germany

## Abstract

The purpose of this study was to assess the effect of three different inoculation routes into mycoplasmal pneumonia (MP) in pigs challenged with *Mycoplasma hyopneumoniae* (*M. hyopneumoniae*). Thirty six-week-old *M. hyopneumoniae* seronegative piglets were randomly assigned to four groups: three challenged groups with experimentally inoculated pigs by either the endotracheal (ET; *n* = 8), intranasal (IN; *n* = 8) or aerosol (AE; *n* = 8) routes and one uninfected group (Control; *n* = 6). Blood samples were collected 1 day before challenge and at necropsy, 28 days post-inoculation (dpi), to assess seroconversion. Laryngeal swabs were collected at −1, 7, 14, 21 and 28 dpi in order to evaluate colonization. At necropsy, lung lesions were scored and lung tissue was collected for histopathological studies and *M. hyopneumoniae* DNA detection. Broncho-alveolar lavage fluid (BALF) was also obtained to detect *M. hyopneumoniae* DNA, specific IgA antibodies and cytokines. MP was observed in all inoculated groups, but the ET group displayed a significantly higher number of animals affected by MP as well as a higher mean lung lesion score. These results were paralleled with an earlier seroconversion and upper respiratory tract colonization of *M. hyopneumoniae*. Additionally, in the ET group, higher levels of pro-inflammatory cytokines and specific IgA antibodies in BALF were found. Under the conditions of the present study, MP was reproduced by the three evaluated inoculation routes. Obtained results suggest that the ET route is the most effective in order to induce MP in pigs experimentally challenged with *M. hyopneumoniae*.

## Introduction

*Mycoplasma hyopneumoniae* (*M. hyopneumoniae*) is the causal agent of mycoplasmal pneumonia (MP). MP is frequently complicated with other bacteria (enzootic pneumonia [EP]) and viruses (porcine respiratory disease complex [PRDC]), which affect the severity of the disease. These chronic respiratory infectious processes affect mainly growing and finishing pigs and are significant causes of economic losses to swine producers throughout the world [1].

*M. hyopneumoniae* experimental models are essential for vaccine efficacy assessment. However, optimal and reproducible results are not always achieved. In fact, variation in pneumonic lesion severity has been reported in different experimental *M. hyopneumoniae* challenge systems [2–6], also among animals challenged with the same isolate and dose [3, 6, 7]. Some of the aspects that may influence the *M. hyopneumoniae* infection pattern and the severity of the associated lung lesions are the strain, duration of the study, type and dose of the inoculum and the inoculation route. The intrinsic virulence of *M. hyopneumoniae* strains has been demonstrated to determine the clinical course of the infection [6, 8]. Days lapsed between challenge and sacrifice (duration of the study) have been related to clinical signs appearance and lung lesions development [4, 5, 9, 10]. In addition, the host immune response to infection is considered a major driver of lung pathology, although the underlying inflammatory mechanisms are not yet well understood [10, 11]. With regard to the latter factor, the use of lung homogenate instead of pure culture as inoculum might interfere with the observation of any specific response to *M. hyopneumoniae* due to the inflammatory response caused by the administration of foreign antigens [12].

One of the most distinguishing features of experimental challenge systems is the *M. hyopneumoniae* inoculation route used. However, its impact on the pathogenesis of the experimentally induced *M. hyopneumoniae* infection has been poorly investigated. There are four inoculation routes reported in the peer-reviewed literature used in swine *M. hyopneumoniae* challenge models: endotracheal (ET), transtracheal (TT), intranasal (IN) and aerosol (AE). Although both intratracheal methods (ET and TT) are the most widely used, MP has been induced by all models. AE is probably the least extended method, despite it is supposed to mimics better the natural conditions of infection [12]. Comparisons between *M. hyopneumoniae* challenge models using different inoculation routes are scarce. Marois et al. [7] compared the ET, the TT and the IN routes, but no differences regarding detection and recovery of *M. hyopneumoniae*, clinical signs and lesion scores were evidenced between infection conditions.

This last point should be subjected to further investigation. Therefore, the aim of this study was to compare three *M. hyopneumoniae* inoculation routes (ET, IN and AE) for their ability to induce MP. The optimum inoculation route was established by studying colonization, clinical, pathological and immunological parameters.

## Materials and methods

### Animals and housing

Animals were obtained from a herd located in North-Eastern Spain that was free from *M. hyopneumoniae* and *Porcine reproductive and respiratory syndrome virus* (PRRSV) based on serology and clinical history. For animal selection, serology (IDEIA™ *Mycoplasma hyopneumoniae* EIA kit; Oxoid, UK) and a nested PCR (nPCR) for detection of *M. hyopneumoniae* DNA [13] was done on nasal swabs. Thirty four-week old piglets were selected and transported to the experimental facilities of A.M. Animalia Bianya S.L. (Girona, Spain). Prior to challenge, animals were randomly distributed (Randbetween function of Excel 2007 software, Microsoft Office^®^) into four groups equalled according to body weight. Challenged animals were comingled in the same room whereas the control group was placed in a separated room.

### Experimental design

At approximately 6 weeks of age, pigs were challenged according to the experimental design detailed in Table 1. All animals belonging to the challenged groups (*n* = 24) were inoculated with 5 mL of *M. hyopneumoniae* fresh culture on two consecutive days. Two control animals received 5 mL of sterile phosphate buffered saline (PBS) on two consecutive days by one of the three assessed routes (total of *n* = 6). At 28 days post-inoculation (dpi), all pigs were euthanized with an intravenous overdose of sodium pentobarbital and subjected to necropsy examination.

**Table 1:** Experimental design

Group name	No. of animals	Route of inoculation	Inoculum	
			1^st^ day of challenge (0 dpi)	2^nd^ day of challenge (1 dpi)
Control	2	Endotracheal	5 mL PBS	5 mL PBS
	2	Intranasal		
	2	Aerosol		
ET	8	Endotracheal	5 mL *M. hyopneumoniae* fresh culture	5 mL *M. hyopneumoniae* fresh culture
IN	8	Intranasal		
AE	8	Aerosol		

The inoculation took place in 2 consecutive days (0 and 1 dpi). All pigs were euthanized at 4 weeks after the first inoculation (28 dpi).

Study procedure was approved by the Animal Experimentation Ethics Committee of the Universitat Autònoma de Barcelona (n°= 5796) and of A.M. Animalia Bianya S.L. (n°= 05/15).

### Inoculum and inoculation procedures

A fresh culture derived from a *M. hyopneumoniae* field strain was used as the inoculum. This strain was isolated in 2010 from a lung of a slaughter-age animal showing MP. Inoculum titre was determined by using a limiting dilution method. Briefly, ten-fold dilutions of the inoculum were made and left to grow for 2 weeks at 37 °C. Tubes were tested for *M. hyopneumoniae* by PCR [14] at 1 and 2 weeks of incubation in order to indirectly evaluate bacterial growth. From the 2nd week PCR results, the final titre was calculated by means of the Reed and Muench method [15]. The inoculum titre was 8.25 log10 PCR_50_/mL, in which PCR_50_ represents the limiting dilution of the inoculum that is PCR positive in 50% of its replicates.

For the ET inoculation, a double catheter (an internal with a syringe adapter into an external catheter) (Bastos Medical S.L., Spain) was introduced in the trachea. The inoculum was administered with a syringe through the internal catheter. For the IN inoculation, a mucosal atomization device (MAD Nasal™; Wolfe Tory Medial, Inc., USA) attached to a syringe was used to administrate half of the inoculum volume into each nostril. Animals included in the AE group, were anaesthetised with a combination of 10 mg/kg Ketamine (Imalgene^®^; Merial, France), 0.4 mg/kg Butorfanol (Torbugesic^®^-SA; Zoetis, USA) and 6 mg/kg of Azaperone (Stresnil^®^; Esteve, Spain) and placed in sternal recumbence. The inoculum was administered through an individual mask (Bastos Medical S.L., Spain) connected to an aerosol delivery system (Boy^®^ SX compressor and LC^®^ Sprint nebulizer; Pari GmbH, Germany) with a total output rate of approximately 600 mg/min and a particle mass median diameter of 3.5 µm under a pressure of 1.6 bar.

### Clinical evaluation and body weight

After inoculation, pigs were monitored for clinical signs on a weekly basis for 4 weeks. The focus of clinical observations was on respiratory signs such as dyspnoea and coughing. Body weight was registered prior to the challenge and at necropsy day. Average daily weight gain (ADWG) was calculated according to the following formula: body weight at necropsy minus the body weight before challenge divided by the days lapsed between them.

### Collection and samples processing

Blood was collected 1 day before challenge (−1 dpi) and at necropsy day (28 dpi). Laryngeal swabs were obtained as described previously [16] at −1 dpi and weekly thereafter (7, 14, 21 and 28 dpi). Once in the laboratory, blood was centrifuged at 1500* g* for 10 min at 4 °C and sera were stored at −80 °C until used. Laryngeal swabs were resuspended in 1 mL sterile PBS, vortexed and stored at −80 °C.

At necropsy (28 dpi), two lung samples were collected from all each animals: one was fixed in 10% neutral buffered formalin and the second one was frozen. These samples were used for histopathological studies and detection of *M. hyopneumoniae* DNA respectively. Broncho–alveolar lavage fluid (BALF) was collected from twelve animals, selecting those three animals showing the most severe lung lesions (when present) within each group. BALF was left for gross mucus sedimentation and supernatant was stored at −80 °C until it was processed. These samples were used for *M. hyopneumoniae* DNA, cytokine and specific IgA antibody detection.

### Pathological examination

The extension of gross lung lesions compatible with *M. hyopneumoniae* infection (cranio-ventral pulmonary consolidation; CVPC) was assessed using the European Pharmacopoeia (Ph. Eur., monograph no. 04/2013:2448) scoring system. For histopathological studies, formalin-fixed tissues were processed routinely and embedded in paraffin wax. Sections (4 μm) were stained with haematoxylin and eosin and examined under light microscope for broncho-interstitial pneumonia (BIP). Microscopic scoring was performed as previously described [17]. Briefly, histopathological lung lesions were graded from 0 to 4, where 0 to 2 was classified as non-compatible with MP and 3 to 4 was considered compatible with MP microscopic lung lesions.

### Detection of *M. hyopneumoniae*-specific antibodies in serum and BALF

Sera were tested in duplicate for *M. hyopneumoniae* antibodies by means of a commercial competitive inhibition enzyme-linked immunosorbent assay (IDEIA™ *Mycoplasma hyopneumoniae* EIA kit; Oxoid, UK). Samples with mean optical density (OD) <50% of the OD of the buffer control were considered positive. Doubtful (OD from 50 to 64%) and negative (OD ≥ 65%) OD-values were classified as negative in the statistical analysis.

Detection of *M. hyopneumoniae* specific IgA in BALF was performed modifying the *Mycoplasma hyopneumoniae* Antibody Test Kit (BioCheck, UK) with an alkaline phosphatase-labelled goat anti-porcine IgA polyclonal antibody (Bethyl Laboratories, USA) at 1:5000 dilution as secondary antibody. BALF samples were tested undiluted and in duplicate. One hundred µL of each sample were used in the ELISA assay. The cut-off was established at mean OD value of BALF from the control animals plus three folds the standard deviation (SD). Values higher than the cut-off were considered positive. Values below this cut-off were considered negative.

### DNA extraction

DNA was extracted from 200 μL of laryngeal swabs suspension or undiluted BALF using BioSprint^®^ 96 DNA Blood kit (Qiagen GmbH, Germany) on the BioSprint 96 workstation (Qiagen GmbH, Germany). Lung tissue was disrupted using TissueLyser (Qiagen GmbH, Germany) for DNA extraction. Approximately 1 g of tissue was homogenized with 600 µL of PBS into plastic tubes containing glass beads. After shaking for 10 min, the lung homogenate was centrifuged at 11 000 *g* for 1 min. DNA was extracted from 200 μL of tissue supernatant (MagMAX™ DNA Multi-Sample Kit, Life Technologies, USA) according to the manufacturer’s instructions on the BioSprint 96 workstation (Qiagen GmbH, Germany). To assess potential contamination during the extraction procedure, a negative control was included using PBS as an extraction substrate in each extraction plate.

### Real time PCR

A commercial real time *M. hyopneumoniae* PCR (rt-PCR) was performed in laryngeal swabs, BALF and lung tissue samples. The assay was performed using VetMAX™-Plus qPCR Master Mix (Applied Biosystems, USA) and VetMAX™ *M. hyopneumoniae* Reagents (Applied Biosystems, USA), according to the manufacturer’s instructions. VetMAX™-Plus qPCR Master Mix kit includes Xeno™ DNA Control, which serves as an internal positive control for DNA purification and rt-PCR. Runs were carried out in an ABIPRISM^®^ 7500 machine (Applied Biosystems, Singapore). The threshold for the DNA target was set at 10% of the average maximum fluorescence value of the positive control DNA target. Cycle threshold (Ct) values equal to or lower than 40 were considered positive.

### Evaluation of cytokine responses in BALF

Levels of pro-inflammatory cytokines IL-1β, IL-8, IL-6 and TNF-α were determined using a commercially available Porcine Quantikine^®^ ELISA kits (R&D Bio-Scientific Pty Ltd, Australia) following manufacturer’s recommendations. BALF samples were tested undiluted. Reactions were measured using OD at 450 nm and quantified by the use of a standard curve.

### Statistical analyses

Statistical analyses were performed using NCSS software [18]. Normal distribution of continuous variables was evaluated by the Shapiro–Wilk test. An analysis of variance using the Tukey–Kramer test was used for mean comparison of continuous variables (lung score, body weight, ADWG, rt-PCR Ct, cytokine concentrations, percentage of inhibition and IgA ELISA OD) among groups. The Chi square or Fischer tests were applied to evaluate the proportion of animals showing MP, seroconversion and rt-PCR positive results. In order to evaluate the agreement between the rt-PCR results in laryngeal swabs and lung tissue, the Cohen’s kappa coefficient (κ) was calculated. *P* values ≤ 0.05 were considered statistically significant, whereas *p* values > 0.05 and ≤ 0.10 were considered statistical tendencies.

## Results

Negative control pigs were negative for antibodies, clinical signs, DNA and lesions associated to *M. hyopneumoniae* infection throughout the study.

### Clinical signs and body weight

Very sporadic and mild coughing was displayed by challenged pigs from 14 dpi onwards. No other significant clinical signs were observed during the experiment. No significant differences in mean body weight (at challenge and at necropsy) neither in ADWG were observed among groups (data not shown).

### Pathological studies

MP was recognised taking into account three criteria concomitantly: (1) Presence of CVPC, based on gross evaluation, (2) presence of BIP determined through microscopic evaluation (score 3 or 4) and (3) detection of *M. hyopneumoniae* DNA by rt-PCR in lung tissue.

Percentage of animals showing MP and the mean lung score within each group are represented in Table 2. MP was observed in all inoculated groups. No significant differences in the lung lesion score were observed among the IN, AE nor Control groups. In contrast, significant differences were obtained regarding the number of animals showing MP as well as in MP severity between the ET and all the other groups (*p* < 0.05).

**Table 2:** Proportion (%) of animals showing MP and lung lesion score (mean ± SD) at 28 dpi

Group	No. of animals with MP/Total no. of animals (%)	Ph. Eur. score including all animals per group (mean ± SD)	Ph. Eur. score of animals showing MP per group (mean ± SD)
Control	0/6 (0.0)^a^	0.0 (± 0.0)^a^	0.0 (± 0.0)^a^
ET	7/8 (87.5)^b^	9.5 (± 9.8)^b^	10.8 (± 9.8)^b^
IN	2/8 (25.0)^a^	0.9 (± 1.6)^a^	3.6 (± 0.1)^a^
AE	3/8 (37.5)^a^	1.4 (± 2.7)^a^	3.7 (± 3.3)^a^

Different superscripts within a column indicate significant differences between groups (*p* < 0.05).

### Serology and *M. hyopneumoniae*-specific IgA in BALF

**Figure 1:**
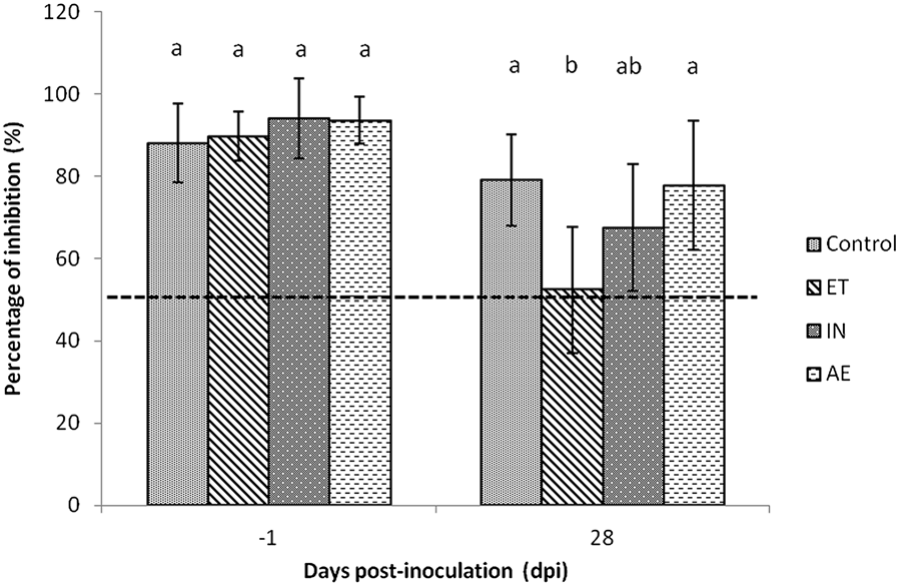
**Sera percentage of inhibition (mean ± SD) from Control, ET, IN and AE groups 1 day before challenge (−1 dpi) and at necropsy (28 dpi)**. Different superscripts indicate significant differences among groups (*p* < 0.05). Discontinuous line represents the seropositivity threshold.

None of the animals was seropositive 1 day before challenge (Figure 1). In both IN and AE groups, only one animal out of 8 (12.5%) seroconverted at 4 weeks after challenge (28 dpi). At that time, five pigs out of 8 (62.5%) seroconverted within the ET group (*p* < 0.05). The ET mean percentage of inhibition was also statically lower (*p* < 0.01) than all the other groups except for the IN. Interestingly, all animals that seroconverted showed MP regardless the experimental group.

**Figure 2:**
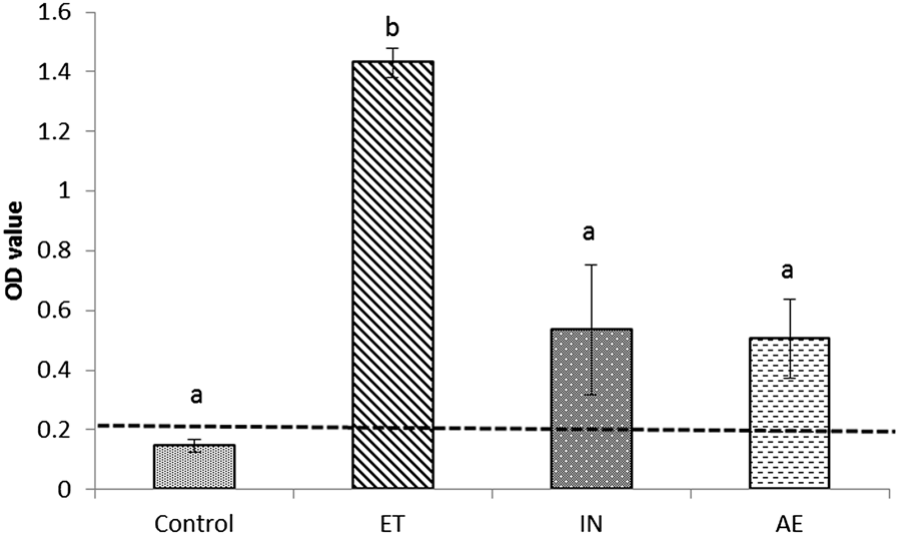
***M. hyopneumoniae*** **-IgA antibodies (mean ± SD of OD values at 405 nm) in BALF from Control, ET, IN and AE groups at 28 dpi**. Different superscripts indicate significant differences among groups (*p* < 0.05). Discontinuous line represents the positivity threshold.

The cut-off value of the indirect IgA ELISA was established at an OD value of 0.21. All BALF samples belonging to the challenged groups (ET, IN and AE) were *M. hyopneumoniae*-IgA positive at 28 dpi at a different levels. The ET group showed a significantly higher (*p* < 0.01) mean OD value than those of the IN, AE and control groups (Figure 2). Although mean specific IgA OD values in BALF from AE and IN groups were not statistically different in comparison with the mean value in Controls, a tendency was reported (*p* ≤ 0.10).

### *M. hyopneumoniae* detection by real time PCR

The percentage of positive animals to *M. hyopneumoniae* rt-PCR and their Ct values (mean ± SD) in laryngeal swabs along the study, in lung tissue and BALF within each group are represented in Table 3.

**Table 3:** Proportion (%) of *M. hyopneumoniae* rt-PCR positive animals in laryngeal swabs through the study, lung tissue and BALF and Ct values (mean ± SD) from Control, ET, IN and AE groups

Groups	Laryngeal swabs										Lung tissue		BALF	
	−1 dpi		7 dpi		14 dpi		21 dpi		28 dpi					
	Prop. (%)	Ct	Prop. (%)	Ct*	Prop. (%)	Ct*	Prop. (%)	Ct*	Prop. (%)	Ct*	Prop. (%)	Ct*	Prop. (%)	Ct*
Control	0/6 (0.0)^a^	NA	0/6 (0.0)^a^	NA	0/6 (0.0)^a^	NA	0/6 (0.0)^b^	NA	0/6 (0.0)^b^	NA	0/6 (0.0)^b^	NA	0/3 (0.0)^b^	NA
ET	0/8 (0.0)^a^	NA	2/8 (25.0)^a^	38.8 ± 0.4	3/8 (37.5)^a^	31.8 ± 2.6	5/8 (62.5)^a^	34.4 ± 1.6^a^	5/8 (62.5)^a^	33.6 ± 2.8^a^	7/8 (87.5)^a^	26.8 ± 0.9^a^	3/3 (100)^a^	24.1 ± 1.0^a^
IN	0/8 (0.0)^a^	NA	0/8 (0.0)^a^	NA	0/8 (0.0)^a^	NA	2/8 (25.0)^ab^	33.1 ± 0.8^a^	6/8 (75.0)^a^	35.3 ± 3.2^a^	5/8 (62.5)^ab^	33.5 ± 6.1^b^	3/3 (100)^a^	22.9 ± 2.2^a^
AE	0/8 (0.0)^a^	NA	0/8 (0.0)^a^	NA	0/8 (0.0)^a^	NA	3/8 (37.5)^ab^	35.4 ± 4.1^a^	3/8 (37.5)^ab^	33.9 ± 5.0^a^	3/8 (37.5)^b^	28.5 ± 7.7^a^	3/3 (100)^a^	26.2 ± 1.9^a^

Different superscripts within a column indicate significant differences between groups (*p* < 0.05).

*Prop* proportion, *NA* non-applicable.

* Mean Ct value has been calculated considering only those rt-PCR positive animals.

All the animals included in the study were negative to rt-PCR in laryngeal swabs before challenge. While in the ET group *M. hyopneumoniae* DNA was detected from 7 dpi onwards, in both IN and AE groups, *M. hyopneumoniae* DNA was not detected until 21 dpi. Independently of the group, all animals that became positive in a certain time point of the study remained positive until the end (28 dpi). The highest percentage of positive animals was detected at 21 dpi in all groups but except for the IN group, in which rt-PCR positive animals appeared to increase over the last week (28 dpi). In fact, the maximum number of positive animals (75%) within a group was present in the IN group at 28 dpi. All the animals showing MP were rt-PCR positive in laryngeal swabs at necropsy day, except two animals belonging to the ET group. No significant differences in mean Ct values were observed between groups at any time point.

*M. hyopneumoniae* rt-PCR positive animals in lung tissue were detected in all challenged groups, but the highest percentage of positive animals belonged to the ET one. All animals with MP were rt-PCR positive in lung tissue at necropsy day regardless of the group. However, three animals without MP from the IN group were also lung rt-PCR positive. From the total of the 15 animals that were *M. hyopneumoniae* positive in lung tissue, 11 were also positive in laryngeal swabs at the same time point of the study (28 dpi), showing both techniques a coincidence of 77% (κ = 0.53). Although in the IN group 5 out of 8 animals (62.5%) were rt-PCR positive, mean lung tissue Ct value was significantly (*p* < 0.05) higher in this group compared to the other two challenged groups.

All the BALF samples from the three challenged groups (ET, IN and AE) were rt-PCR positive. No significant differences in mean Ct values were observed in BALF between groups.

### Cytokine responses in BALF

**Figure 3:**
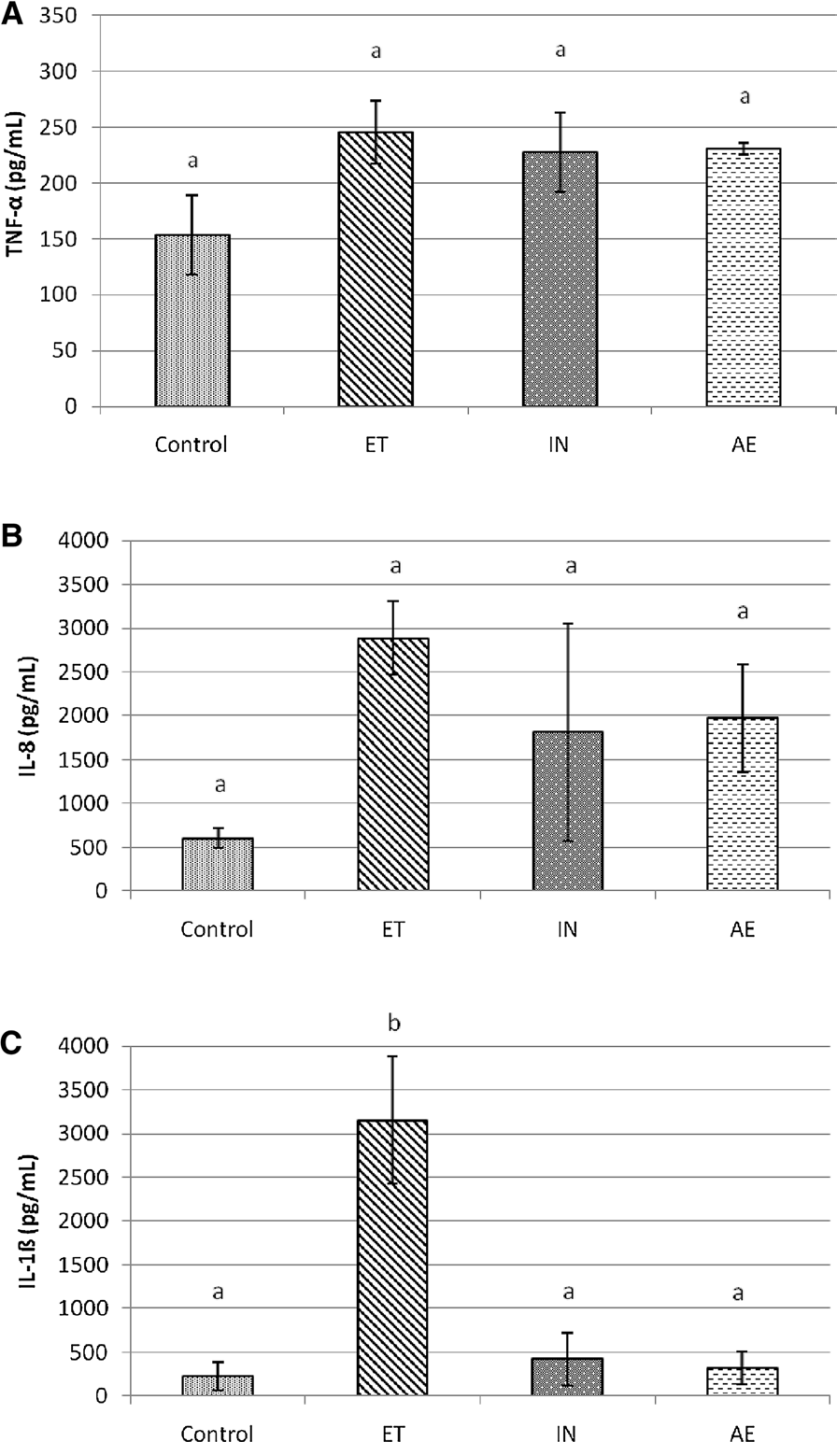
**Levels (mean ± SD) of TNF-α (** **A** **), IL-8 (** **B** **) and IL-1β (** **C** **) in BALF of pigs from Control, ET, IN and AE at 28 dpi**. Different superscripts indicate a significant difference among groups within each graph (*p* < 0.05).

Four weeks after infection, IL-6 cytokine was not detected in any of the BALF samples tested (data not shown). Mean TNF-α (Figure 3A) and IL-8 (Figure 3B) cytokine levels were not significantly different between groups. Nevertheless, there was a tendency between mean levels of TNF-α and IL-8 from ET group and those found in Controls (*p* ≤ 0.10). Remarkably, mean levels of IL-1β (*p* < 0.001) increased significantly in ET-challenged pigs in comparison with mean levels found in all other groups (Figure 3C).

## Discussion

The ability to induce MP in 6-week-old conventional piglets experimentally challenged with *M. hyopneumoniae* using three different inoculation routes was assessed in the present study. Lung lesion scores have been previously reported to be similar between groups of pigs experimentally challenged by means of different inoculation routes [7]. However, different *M. hyopneumoniae* infectious doses and necropsy timings were used, difficulting comparisons with the present study, as such factors are known to influence the infection pattern and MP severity in pigs [5]. To the best of author’s knowledge, this is the first time that the ET, the IN and the AE inoculation routes are compared under the same conditions in pigs challenged with *M. hyopneumoniae*.

The three tested inoculation routes (ET, IN and AE) had been already reported to induce MP [6, 7, 12, 19]. In agreement with these previous studies and under the settings applied in the present study, all inoculation route models reproduced MP. However, the highest percentage of animals affected and the most severe lung lesions were obtained in the ET inoculated group. These results were accompanied by an earlier respiratory tract colonization and seroconversion, a higher percentage of rt-PCR positive animals in lung tissue and an enhanced cellular and humoral local immune response. On the other hand, no significant differences in clinical signs neither in mean body weight at necropsy nor in ADWG were observed between groups. The number of animals used, frequency of clinical signs observation (only weekly) and duration of the study period were probably too limited to observe any effect of the inoculation route on coughing and performance parameters.

*M. hyopneumoniae* is primarily found on the mucosal surface of the trachea, bronchi and bronchioli [20], and its adherence to the ciliated epithelium is a prerequisite for initiation of the infection [21]. Previous studies indicate that tracheo-bronchial swabbing is the most sensitive sampling method for detecting *M. hyopneumoniae* in infected live pigs using nested or real-time PCR assays [7, 22, 23]. The use of laryngeal swabs to assess *M. hyopneumoniae* infection has been recently reported [16]. Under the present conditions and despite being ante-mortem and post-mortem samples respectively, 77% of concordance (κ = 0.53) between laryngeal swabs and lung tissue for *M. hyopneumoniae* detection by rt-PCR was found at necropsy. In the present report, colonization timing differences were found between groups; *M. hyopneumoniae* DNA was detected in the ET group 2 weeks earlier (7 dpi) than in the IN and AE groups (21 dpi). Although only two out of eight (25%) IN-challenged animals were showing MP, laryngeal swabs rt-PCR positive animals appeared to increase over the last week (28 dpi) in this group, accompanied by quite a high proportion (5 out of 8) of rt-PCR positive animals in lung tissue at that time. It has been reported that *M. hyopneumoniae* gross lung lesions reached their maximum score by 28 dpi [4, 5, 9, 10]. In consequence, in most experimental infection models, pigs are euthanized at this point. Nonetheless, it cannot be ruled out that, in AE and/or IN-challenged animals, longer study periods would have increased the percentage of animals showing MP and its severity.

One limitation of this study was the fact that challenged animals were comingled in the same room and transmission between groups was possible throughout the study. In consequence, whether the infection observed in IN and AE groups is product of the ET *M. hyopneumoniae* shedding cannot be completely excluded. However, this event would be very unlikely since the first evidence of *M. hyopneumoniae* infection was seen at 7 dpi on two animals from the ET group. Considering that necropsies were done 3 weeks after this first detection, the probability that seroconversion and lung lesions observed in AE and IN-challenged animals were due to these two pigs is probably very low.

Among many other factors, development of MP is dependent on the number of organisms that colonize the respiratory tract, which is likely dependent on cumulated infectious doses [1]. Since all groups were challenged with the same isolate and dose, the present results point out that the inoculation route may have a certain impact on the survival and/or infectivity of *M. hyopneumoniae*, which then is reflected on its colonization and in consequence, on the infection outcome. Information regarding how the inoculation route can affect the survival and infectivity of *M. hyopneumoniae* particles is limited. By means of the ET route, the inoculum penetration into the lower respiratory tract is expected to be higher, which might enhance colonization. Although IN and, more importantly, the AE inoculation routes mimic better the natural infection conditions, the pig’s long and curving respiratory tract may represent a major barrier for such administrations [24]. Related to this, the size of the aerosol particles is relevant, because it influences the time until they settle as well as the depth of penetration in the respiratory tract upon inhalation [25]. While *M. hyopneumoniae* bacterium mean diameter is 0.20 µm [26], deposition of aerosols in trachea and bronchiole was maximal when aerosol droplet size was 2–5 μm [24, 27], a range that includes the mean particle size used in the present study (3.5 μm). On the contrary, the mucosal atomization device used in the IN group sprays a fine mist of particles of 30–100 μm in size. Although the important variation between particles sizes derived from both devices, in this study no difference in the ability to induce MP was evidenced between the IN and AE inoculation conditions. In fact, a higher colonization was recorded in the IN group at 28 dpi, meaning that other conditions rather than size of particles might influence the *M. hyopneumoniae* deposition in the respiratory tract.

In the current study, a significantly earlier seroconversion and lower percentages of inhibition were found in the ET group. The reason for this finding might be due to the higher cumulated infectious dose achieved via the ET inoculation route, which might imply a faster colonization of *M. hyopneumoniae* and, therefore, a higher exposure to the mucosal immune system. In fact, all animals that seroconverted at necropsy had also MP. Indeed, it is well known that the concentration of serum antibodies does not correlate with clinical protection against *M. hyopneumoniae* [28, 29]. Nevertheless and similarly to serum results, significantly higher specific IgA levels in BALF were detected in the ET group. Although specific locally secreted IgA after vaccination has been postulated to play a pivotal role in preventing MP development [30–33], the results obtained are in accordance to the results reported by Djordjevic et al. [28], where IgA antibody concentrations achieved after *M. hyopneumoniae* challenge in BALF did not prevent MP development. Since the three BALF samples that were taken per group belonged to those animals with the highest lung scores (when MP was present) and comparisons with those animals without lesions within a group could not be performed, BALF results are likely overestimated.

The evaluation of the mucosal immune response was further addressed by the analysis of cytokine levels in BALF. Cytokine responses in BALF from control animals were found to be low and within a clinically normal range by comparing with previously reported data [6, 10, 32]. Increased levels of the pro-inflammatory cytokines in BALF from *M. hyopneumoniae* infected pigs [6, 10, 32, 34, 35] and their relationship with the occurrence of pneumonic lesions [3, 36] have already been reported. However, the present study aimed to determine whether there was a measurable difference in levels of these cytokines between pigs challenged by different inoculation routes. Although possible overestimation due to the abovementioned reason, IL-1β response was significantly more prominent in the ET group than in the IN and AE challenged groups 4 weeks after infection. As reported in this study, high BALF levels of IL-1β are associated with tissue damage in the early stages of the infection [6, 10, 34]. In contrast, the lower levels of the IL-8 and, more importantly, of IL-1β in BALF of the IN and AE inoculated animals in comparison with the ones from the ET group, also support the pathological data, since inflammation was significantly lower in these two groups (IN and AE).

Under the conditions of this study, ET inoculation route was more effective inducing MP 4 weeks after challenge than the IN or AE ones. ET route is expected to apply a greater inoculum volume in pig’s lower respiratory tract, achieving greater infectious doses in shorter times and promoting an earlier *M. hyopneumoniae* colonization and immune response against infection, which at the end is reflected in a higher incidence and more severe MP.

## Abbreviations

ADWG: average daily weight gain
AE: aerosol
BALF: broncho-alveolar lavage fluid
BIP: broncho-interstitial pneumonia
Ct: cycle threshold
CVPC: cranio-ventral pulmonary consolidation
ELISA: enzyme-linked immunosorbent assay
EP: enzootic pneumonia
ET: endotracheal
Ig: immunoglobulin
IL: interleukin
IN: intranasal
*M. hyopneumoniae*: *Mycoplasma hyopneumoniae*
MP: mycoplasmal pneumonia
OD: optical density
PBS: phosphate buffered saline
PCR: polymerase chain reaction
Ph. Eur.: European Pharmacopoeia
PRDC: porcine respiratory disease complex
PRRSV: porcine reproductive and respiratory syndrome virus
Rt-PCR: real time PCR
TNF: tumor necrosis factor
TT: transtracheal
